# Improved Detection of HIV Gag p24 Protein Using a Combined Immunoprecipitation and Digital ELISA Method

**DOI:** 10.3389/fmicb.2021.636703

**Published:** 2021-03-16

**Authors:** Guoxin Wu, Carol Cheney, Qian Huang, Daria J. Hazuda, Bonnie J. Howell, Paul Zuck

**Affiliations:** Department of Infectious Disease and Vaccines, Merck & Co. Inc., Kenilworth, NJ, United States

**Keywords:** HIV, p24, SIV, p27, immunoprecipitation, Simoa, rectal biopsy, biomarker

## Abstract

Greater than 90% of HIV-1 proviruses are thought to be defective and incapable of viral replication. While replication competent proviruses are of primary concern with respect to disease progression or transmission, studies have shown that even defective proviruses are not silent and can produce viral proteins, which may contribute to inflammation and immune responses. Viral protein expression also has implications for immune-based HIV-1 clearance strategies, which rely on antigen recognition. Thus, sensitive assays aimed at quantifying both replication-competent proviruses and defective, yet translationally competent proviruses are needed to understand the contribution of viral protein to HIV-1 pathogenesis and determine the effectiveness of HIV-1 cure interventions. Previously, we reported a modified HIV-1 gag p24 digital enzyme-linked immunosorbent assay with single molecule array (Simoa) detection of cell-associated viral protein. Here we report a novel p24 protein enrichment method coupled with the digital immunoassay to further extend the sensitivity and specificity of viral protein detection. Immunocapture of HIV gag p24 followed by elution in a Simoa-compatible format resulted in higher protein recovery and lower background from various biological matrices and sample volumes. Quantification of as little as 1 fg of p24 protein from cell lysates from cells isolated from peripheral blood or tissues from ART-suppressed HIV participants, as well as simian–human immunodeficiency virus–infected non-human primates (NHPs), with high recovery and reproducibility is demonstrated here. The application of these enhanced methods to patient-derived samples has potential to further the study of the persistent HIV state and examine *in vitro* response to therapies, as well as *ex vivo* study of translationally competent cells from a variety of donors.

## Introduction

Antiretroviral therapy (ART) has dramatically improved life quality and survival for people living with HIV (PLWHs). Despite long-term ART, HIV persists and viremia almost invariably rebounds once therapy is withdrawn (Li et al., 2016; Castagna et al., 2019; Cohn et al., 2020). Several HIV cure strategies are being employed to eradicate the virus including latency-reversing agents, broadly neutralizing antibodies, therapeutic vaccines, and immune-based approaches (Thorlund et al., 2017; Sobieszczyk, 2020). Cure-directed interventions will require well-characterized clinical studies to inform outcomes, as well as appropriate biomarkers to establish proof-of-mechanism and sensitive measurement of both the active and latent HIV reservoir in blood and tissue compartments wherever possible. Biomarkers that can robustly measure viral persistence in PLWHs on ART in a way that reports on proviruses capable of driving rebound off therapy or producing viral protein that influence HIV pathogenesis and immune functions are important tools for more in-depth understanding of HIV persistence and assessment of therapeutic interventions.

Recent studies of viral persistence have relied on differentiating between replication-competent and defective provirus (Bruner et al., 2016; Falcinelli et al., 2019). The quantitative viral outgrowth assay (QVOA) measures the replication-competent HIV reservoir but likely underestimates reservoir size as some genetically intact viruses may not grow in culture after a single round of *ex vivo* stimulation. Limitations also include cost, assay duration, requirement for large cell numbers, throughput, and relying primarily on cells obtained from blood (Crooks et al., 2015; Stone et al., 2020; Stuelke et al., 2020). HIV DNA quantification is a simple, standardized, sensitive, and reproducible assay but lacks specificity for replication-competent proviruses (Avettand-Fènoël et al., 2016). The newer intact proviral DNA assay (Bruner et al., 2019) shows promise and requires relatively fewer cells than QVOA, but the predictive value in measuring changes in reservoir size with cure-directed interventions remains unknown. HIV RNA assays quantify transcriptionally active proviruses but fail to distinguish between intact and truncated proviruses leading to inflated reservoir sizes (Cillo et al., 2014; Pasternak and Berkhout, 2018) and do not inform on whether RNA transcripts produce antigen or virions capable of spreading infection or shaping immune responses. Studying viral protein is becoming increasingly important for cure research as proteins are more likely than nucleic acid to be sensed by the immune system, influence immune functions, and can be produced by some defective proviruses (Imamichi et al., 2020). Although protein-based assays also overestimate replication competent reservoir size, measuring this translationally competent reservoir provides mechanistic insight into HIV persistence, immune response, and evaluation of interventional strategies aimed at clearing these infected cells.

High-sensitivity HIV gag p24 assays have emerged in recent years including the digital enzyme-linked immunosorbent assay (ELISA) or single-molecule array (Simoa). The Simoa approach has shown several logs improvement in p24 detection in plasma and serum over traditional immunoassays (Chang et al., 2013; Passaes et al., 2017). Previously, we first reported use of the Simoa assay for the specific measurement of cell-associated p24 in CD4^+^ T cells isolated from peripheral blood of ART-suppressed HIV^+^ individuals following *ex vivo* or *in vivo* HIV latency reactivation (Wu et al., 2017) and recently extended the methodology to tissues (Wu et al., 2021). Despite these improvements, additional factors may impose challenges in sensitive detection of low-abundant analytes and may lead to underreporting actual analyte concentrations in biophysiological sample types. For example, sample matrix can interfere with assay signal generation due to the concentration and viscosity of the matrix (Wood, 1991). Sample dilution may help to overcome this interference but might not be feasible if high sample concentration is needed for detecting low-abundance proteins. Similar challenges exist for detecting HIV p24-producing cells as these are rare in a population of cells (Graf et al., 2013; Deeks et al., 2016) requiring larger cell numbers to have relatively few p24-producing cells. The challenge in this situation is the balance between concentrated matrix proteins in a lysate of large cell numbers vs. overly dilute analyte. To overcome this, we developed a novel approach to allow for larger cell input numbers while minimizing matrix effects for sensitive and specific measurement of p24 protein from biological samples. A new immunoprecipitation (IP) application was developed for the capture, concentration, and elution of HIV gag p24 protein followed by sensitive measurement using the Simoa p24 assay. We showed greater than 98% recovery of recombinant protein spiked into cell lysates, as well as enhanced p24 measurement from blood-derived CD4^+^ T cells stimulated *ex vivo* with strong or weak latency-reversing agents to produce p24 and detection of p24 from lymphoid tissue (rectal pinch biopsy) isolated from HIV^+^ viremic and aviremic donors. The methods are applicable to HIV p24 as well as SIV p27 proteins and will play a unique role for viral protein detection in cure research beyond the existing nucleic and protein-based assays.

## Materials and Methods

### Participants

Peripheral blood and rectal tissue from HIV-infected subjects were obtained under institutional review board (IRB) approval and patient informed consent through iProcess Global Research (Dallas, TX), University of Pennsylvania (Philadelphia, PA), or internal clinical studies at Merck & Co., Inc. (Kenilworth, NJ). HIV-negative rectal tissue samples were sourced through BioIVT as fresh-frozen tissues taken from healthy adjacent tissues from surgical procedures or postmortem donors. The HIV-negative samples were cut while frozen into small pieces to use in place of HIV-negative pinch biopsies.

### Human CD4^+^ T Cell Separation From Peripheral Blood Mononuclear Cell and Treatment

CD4^+^ T cells were isolated from peripheral blood mononuclear cells (PBMCs) with EasySep^TM^ Human CD4^+^ T Cell Enrichment Kit from StemCell Technologies (Vancouver, BC, NJ) in accordance with the manufacturer’s instructions. Cell numbers and viability were determined using a Vi-cell (Beckman Coulter, Brea, CA). To reactivate virus, 4 × 10^6^ CD4^+^ T cells were incubated with either Dynabeads^TM^ human T-activator anti-CD3/anti-CD28 beads at 25 μL per million CD4^+^ T cells or final 1 μM histone deacetylase inhibitor (HDACi) vorinostat (VOR) in 24-well plates containing 1 mL cRMPI/10 U/mL interleukin 2 for 3 days at 37°C, 5% CO_2_. Cells were collected and lysed at 4 × 10^6^ cells/mL with Simoa lysis buffer [1% Triton X-100, 50% Hi-FBS, and 50% casein/phosphate-buffered saline (PBS)] for 30 min with 1 × protease inhibitor cocktail. Culture medium (CM) was also collected and inactivated with 1% Triton-X100 (final concentration). Samples were stored at -80°C until IP or direct p24 measurement. For cell killing experiments, final 1 μM staurosporine was added together with anti-CD3/anti-CD28 beads.

### Conjugation of Anti-p24 Antibody to Magnetic Beads

Anti-p24 monoclonal antibodies obtained from Capricorn (Portland, ME), ZeptoMetrix (Buffalo, NY), US Biological (Salem, MA), and R&D systems (Minneapolis, MN) were conjugated to magnetic beads (Thermo Fisher Scientific) according to manufacturer’s instructions. In brief, 1 mg/mL antibody (600 μg)/PBS was mixed with 60 mg magnetic beads in kit conjugation buffer for 16 h, 37°C, with rotation. Using magnetic bead capture, beads were washed, blocked, and resuspended in 6-mL storage buffer and stored at 4°C until use. Assuming complete capture of antibody onto the beads, the final antibody concentration is estimated to be 100 μg/mL in the storage solution. Mouse immunoglobulin G (IgG) (mIgG; GenScript) was also used as negative control antibody for conjugation.

### IP of HIV p24 Protein

Frozen lysates from CD4^+^ T cells and rectal biopsies were thawed in 37°C water bath and spun at 20,000 × *g* for 10 min to remove cell debris. Four micrograms antibody-conjugated beads {40 μL beads was washed once with 1 mL of bead washing buffer [PBS + 0.5% bovine serum albumin (BSA)] prior to adding} was added to clarified supernatant. An equal volume of 3% BSA/PBS was added into the inactivated CM and above cell lysate supernatant to yield final 0.5% Triton X-100 in IP mixture. Cell lysate concentration was equivalent to 2 × 10^6^/mL. The IP mixture was incubated at 4°C overnight with gentle rocking. The next morning, the supernatant (“flow-through”) was removed, and beads were washed twice with 1 mL bead washing buffer in 1.7 mL Eppendorf tubes using a magnetic rack to immobilize the beads. Then the bound p24 protein was eluted with 100 μL of 0.1% trifluoroacetic acid (TFA) elution buffer after mixing in a 37°C water bath for 30 min. The eluate was collected, and the beads were washed once with 100 μL of Simoa lysis buffer containing 1 μL of 1 M, pH 9.0, Tris buffer (1:100 dilution, prepared fresh) and mixed, and residual p24 eluate was combined with the initial eluate (total 200 μL) to maximize recovery, reduce non-specific binding, and neutralize the pH for the subsequent measurement. Samples were spun at 20,000 × *g* for 5 min again before p24 Simoa assay, and the supernatant was collected with magnet to ensure no beads from the IP (2.8 μm in diameter) were carried over into the eluate to minimize potential interference with the Simoa assay. Other elution reagents including 0.1 M glycine pH at 2.5, 0.1 N HCl, 0.1 M citric acid, or heat treatment of the beads after IP were compared for their elution efficiency, recovery, and detection of p24. Additionally, the volume of beads used for IP, sample IP incubation duration, and the impact of protease inhibitor cocktail on p24 IP recovery were also examined (see section “Results”) for optimizing IP conditions.

### Culture Media and Cell Lysate Precleaning

Frozen CM and lysate were thawed at 37°C and centrifuged at 20,000 × *g* for 10 min at 4°C. The supernatants were collected after spinning and precleaned with 40 μL of Dynabeads^TM^ M-280 Streptavidin beads from Thermo Fisher containing final 10 μg/mL mIgG (this is to preabsorb any proteins that may stick non-specifically to the beads in the Simoa assay). The mixture was incubated at 4°C for 3 h with rotation using a HulaMixer, and then the lysate was spun at 20,000 × *g* for 10 min. The precleaned supernatant was collected for Simoa measurements (no IP).

### Direct Lysis of Rectal Biopsy

Rectal pinch biopsy samples from HIV^+^ donors or HIV-negative rectal tissue samples (see section “Participants”) were lysed directly by putting the tissue into 0.5 mL of 1% Triton X-100 in Simoa lysis buffer containing 30 μL of uncoated Dynabeads^TM^ M-280 streptavidin beads (for precleaning) in 1.7 mL Eppendorf tube overnight at 4°C with rotation on HulaMixer. The lysate was spun at 20,000 × *g* for 10 min and put on the magnet to hold the beads, and the supernatant was collected. In addition, the pellet was washed once with 0.5 mL of 3% BSA/PBS, spun, and put onto the magnet. The supernatant was collected and combined with the above 0.5 mL lysate supernatant to collect any residual p24 protein remaining in the pellet. The combined yields 1 mL lysate with final 0.5% Triton X-100, which is ready for p24 IP.

### CD4 Protein Assay

Anti-CD4 monoclonal antibody from R&D systems (Cat# MAB375-500) was used as capture antibody, and anti-CD4 monoclonal antibody OKT4 from Biolegend (Cat# 317424) was used as detection antibody. OKT4 antibody was conjugated with alkaline phosphatase (AP) according to our previous method (Wu et al., 2011) and sandwich ELISA (Wu et al., 2012) to measure CD4 protein. Luminescent CDP−Star with Sapphire−II Enhancer substrate (T−2214; Applied Biosystems, Foster City, CA) was used as AP substrate (Wu et al., 2008). In brief, 100 μL of 2 μg/mL capture anti-CD4 antibody was coated on 96-microwell plate overnight, washed once with 200 μL PBS with Tween (PBST), and blocked with 200 μL of 3% BSA/PBS for 2 h, and then 100 μL of CD4 standard protein or diluted lysate and 50 μL of 1:500 diluted AP-OKT4 detection antibody in 0.3% Tween 20/3%BSA/PBS were added sequentially and incubated at 4°C overnight with gentle shaking at 30 revolutions/min. The next morning, the plate was washed with PBST six times and developed with AP substrate. The luminescent counts were measured in PerkinElmer EnVision, and the CD4 concentration was calculated based on the CD4 standard curve. Recombinant CD4 protein obtained from R&D systems was used as assay standard protein stored at −20°C at 1 μM stock concentration in 50% glycerol until using.

### HIV Gag p24 Protein Simoa

HIV gag p24 protein in samples from either precleaned or IP eluant was measured according to our previously published methods (Wu et al., 2017) on the Quanterix HD-X platform. p24 Simoa kit was obtained from Quanterix (Billerica, MA). p24 concentrations were calculated from raw signal average enzyme per bead (AEB) by the instrument’s software with four-parameter logistic regression curve fitting, and p24 levels were reported as pg/million cells or pg/mL in cell lysate and CM (Wu et al., 2017, 2021).

### Simian Immunodeficiency Virus Gag p27 Protein Simoa

Simian immunodeficiency virus (SIV) gag p27 protein was immunoprecipitated with Capricorn anti-p24 antibody conjugated beads as for p24 above. The IP procedure for p27 was the same as above p24. For p27 Simoa after IP and elution, the same kit for p24 was used except that the detection antibody was replaced with biotin-labeled anti-p27 antibody from ABL Inc. (Rockville, MD, Cat# ABL-4324) instead of biotin anti-p24 antibody in the p24 kit. EZ-Link^TM^ Sulfo-NHS-LC-Biotinylation Kit from Thermo Fisher was used for biotinylation of the antibody per the manufacturer’s instruction. Biotin-ABL-4324 antibody concentration used in p27 Simoa was 3 μg/mL in 3% BSA/PBS buffer (Swanstrom et al., 2018). Recombinant p27 protein used for the assay’s standard curve was the gift from Jeff Lifson (Swanstrom et al., 2018). All other assay reagents and parameters were the same as the p24 Simoa conditions. Unknown p27 sample concentrations were calculated based off a p27 assay standard curve.

### Data Analysis

In both p24 and p27 IP-Simoa assay, standards and samples were run in duplicate unless noted otherwise, and unknowns were converted to concentrations by the Quanterix software. Graphs and figures were prepared using GraphPad Prism or Kaleidagraph (Synergy). Statistical significance in group comparisons is denoted conventionally by ^∗^*p* < 0.05, ^∗∗^*p* < 0.01, and ^∗∗∗^*p* < 0.001, using Tukey–Kramer honestly significant difference or Student *t*-test.

## Results

### Development and Optimization of p24 IP Method

**FIGURE 1:**
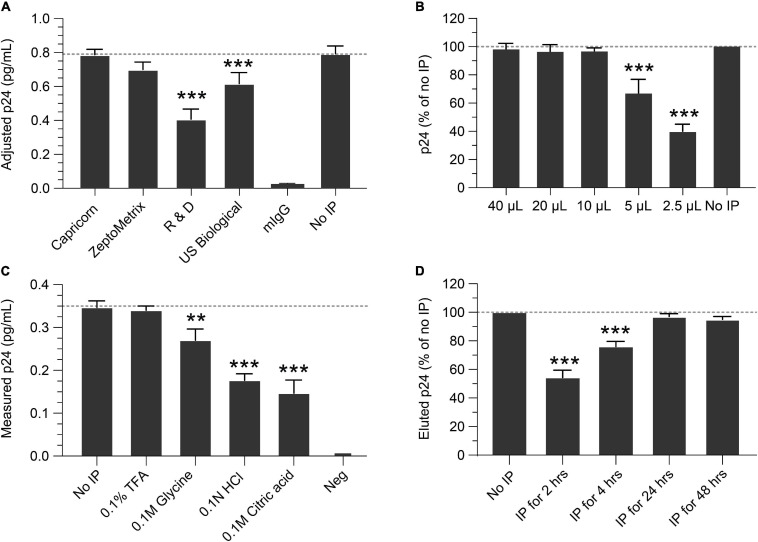
Optimization of the immunoprecipitation (IP) Simoa assay. **(A)** Selection of IP capture antibody. Recovery of p24 using antibodies from various sources, conjugated to magnetic beads, showed the Capricorn antibody to yield the best capture and was similar to the input concentration after release and volume adjustment. **(B)** Optimization of bead concentration for p24 IP. Using a 10 mg/mL stock, adding >10 μL of antibody-conjugated beads to a lysate is sufficient for p24 capture. **(C)** Elution buffer selection. Comparing various elution buffers, 0.1% TFA had no significant difference from input solution after assay, indicating it efficiently releases p24 without destroying crucial epitopes. Other elution buffers resulted in poorer p24 recovery. **(D)** Time required for p24 IP capture. Recovery of p24 with 2- or 4-h incubation with antibody-conjugated beads is markedly lower than that of input solution, whereas recovery with 24 or 48 h gave recovery similar to that of the input solution (no IP). See Materials and Methods for statistical analysis.

IP conditions were evaluated to select the optimal antibody, bead quantity, incubation times, p24 elution buffer, and compatibility with the Simoa assay. To determine the best antibody for IP, four anti-p24 monoclonal antibodies were identified from commercial sources, conjugated to magnetic beads, and compared for IP efficiency and yield of recovery. Based on these criteria, the Capricorn anti-p24 antibody-conjugated beads were selected and used in all following studies (Figure 1A). Using this antibody, we next determined the quantity of antibody-conjugated beads necessary for optimal p24 immunocapture. Antibody-conjugated beads were titrated against a fixed concentration of 1 ng/mL p24. As shown in Figure 1B, 10–20 μL of 10 mg/mL antibody bead solution sufficiently captured 1 ng/mL p24 in 1 mL buffer after overnight incubation at 4°C. To avoid variability in the assay, 40 μL conjugated bead (an excess of the minimal needed) was used for all the following experiments. To identify elution conditions for optimal recovery of p24 bound to beads, the following four buffers were evaluated: 0.1% TFA, 0.1 M glycine, 0.1 N HCL, and 0.1 M citric acid. As shown in Figure 1C, 0.1% TFA yielded the best recovery of p24. Heat denaturation of the complex at 100°C for 10 min resulted in poor protein recovery (data not shown). Based on these data, 0.1% TFA followed by neutralization with Tris was selected for p24 elution and was used in the subsequent experiments. We evaluated the time needed for immunocapture by studying various incubation times of antibody conjugated beads incubated with 1 ng/mL recombinant p24 spiked into Simoa lysis buffer. As shown in Figure 1D, 2- and 4-h incubations resulted in incomplete recovery with 24 and 48 h yielding >95% recovery. Thus, we recommend an overnight incubation (16–24 h) as the optimal time for p24 capture. While no significant impact on p24 recovery was observed following IP overnight with or without protease inhibitor (data not shown), we include it in all experiments to minimize any potential impacts from different samples.

### Capture of Low p24 Concentrations and Assay Validation

**FIGURE 2:**
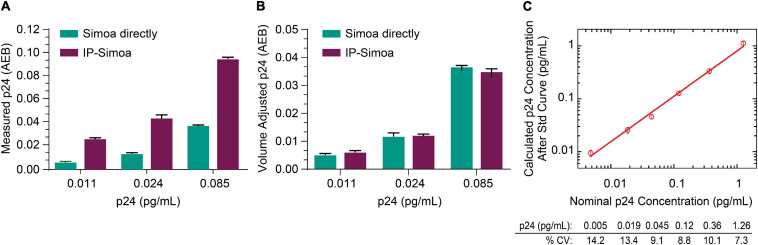
Capture of low p24 concentrations. **(A)** Recombinant p24 at the lowest concentrations of the standard curve was completely recovered after IP, and **(B)** p24 concentration matches between direct Simoa and IP-Simoa after sample volume normalization. **(C)** The IP-Simoa assay’s lower limit of detection (LOD) is ≤0.005 pg/mL defined as the coefficient variation (CV) <20% for each p24 standard. This would equate to 1 fg of p24 concentrated from a given volume and released into 200 μL for assay.

Previously, we optimized the p24 Simoa for detection of protein from cell lysates and found that the assay limit of detection (LOD) was ∼0.005 pg/mL (Chang et al., 2013; Wu et al., 2017). Because of the rare incidence of HIV^+^ cells in biological samples from ART-suppressed patients, we focused efforts on developing IP Simoa methods to quantify very low levels of viral protein (dynamic range ∼0.001–3 pg/mL) and also because high-level protein detection is not an issue for standard, direct Simoa assay (dynamic range of ∼0.01–30 pg/mL). At the lowest point of the p24 standard curve, the signal was approximately 2-fold over background in assay buffer and cell matrix (Wu et al., 2017). To evaluate whether our new IP procedure could boost assay sensitivity and maintain high recovery even at low p24 concentrations, we performed protein immunocapture at the three lowest concentrations in the standard curve (0.011, 0.024, and 0.085 pg/mL). These three standards were mixed with equal volume of HIV-negative CD4^+^ T-cell lysate at 4 × 10^6^/mL in lysis buffer and analyzed in Simoa with or without immunocapture. As shown in Figure 2A, p24 measurements were higher after IP compared to non-IP conditions and proportional to the 3-fold volume reduction from the IP procedure, suggesting near-complete recovery of the p24 from the lysates even at very low analyte concentrations. Figure 2B compares measured p24 values between methods, that is, non-IP conditions and post-IP with accounting for adjustments in sample volume following IP concentration. Assay reproducibility was determined by evaluating the coefficient of variation (CV) for protein standards tested in replicate measurements, and they were found to be very consistent (Figure 2C). LOD was determined by plotting the known concentration of the standard curve with the calculated concentration for the individual measures (Figure 2C). Percent CV was determined for each calculated p24 concentration using the data from the interday runs (*n* = 3), and the threshold for the LOD was set as the lowest standard with CV less than 20%. Based on this criterion, we determined the LOD to be ≤0.005 pg/mL of recombinant p24 standard (Figure 2C). Based on these values and the sample volume input into the Quanterix instrument of 200 μL, we estimate the assay could detect 1 fg of protein concentrated from a given lysate volume to the final detection volume of 200 mL. Of importance, LOD may vary slightly across kits (calibrators, buffers) and instrument settings, and thus investigators should validate this value for their specific conditions.

Using validation samples prepared at 0.4 and 0.2 pg/mL, we showed intraday CV of 11.1 and 8.2% for 0.4 and 0.2 pg/mL, respectively, for IP samples, whereas the interday CVs were 10.4 and 7.4%, respectively. The average recoveries of intraday runs were 86 and 90% for 0.4 and 0.2 pg/mL, respectively (*n* = 3), and the average recoveries from interday runs were 92 and 88% for 0.4 and 0.2 pg/mL, respectively (*n* = 3). We also compared tissue samples from HIV-negative donors and consistently find AEB values indistinguishable from buffer AEB (Supplementary Figure S1). These findings confirm that the immunocapture approach is a robust method for recovering even low amounts of analyte in cell lysates with high reproducibility and shows the potential to enrich p24 from complex matrix, such as cell and tissue lysate.

### Detection of p24 in ART Suppressed HIV^+^ CD4^+^ T Cells

**FIGURE 3:**
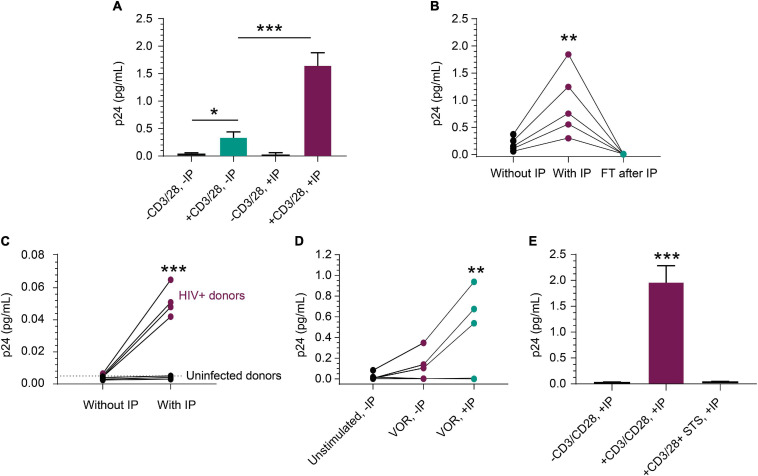
Detection of p24 from HIV^+^, ART-suppressed peripheral blood CD4^+^ T cells. **(A)** p24 production was induced in CD4^+^ T cells following a 3-day stimulation with anti-CD3/anti-CD28 beads (*n* = 3 independent experiments, *p* < 0.05). p24 was enriched 5-fold with a 5-fold volume reduction after IP in treated group (*n* = 3, *p* < 0.001), but not in unstimulated group. **(B)** From samples that respond well to stimulus, p24 was enriched proportionally to volume reduction in five inducible donors (*p* < 0.01), and no detectable p24 was observed in their flow through after IP. **(C)** From samples that do not show robust p24 production after stimulation, IP enrichment was able to demonstrate that p24 is indeed produced but was not at the limit of detection without enrichment. Even with enrichment and bead stimulation, p24 was not detected from HIV-negative CD4^+^ T cells and shows significant difference (*p* < 0.001) between HIV^+^ and uninfected donors after IP. **(D)** p24 protein was detected following a 3-day treatment with 1 μM VOR in three of the five donors’ CD4^+^ T cells (4 × 10^6^ cells/condition). The signal was enriched significantly after p24 IP with 4-fold cell lysate volume reduction (*p* < 0.01). No p24 was detected from unstimulated cells. This opens the door for studying even weak LRAs’ effects using donor-derived cells. **(E)** Mimicking applications with kill-focused assays, p24 was not detected when including 1 μM staurosporine along with anti-CD3/anti-CD28 bead stimulation for those three inducible donors (*p* < 0.001). See Materials and Methods for statistical analysis.

The rare incidence of translationally competent HIV proviruses in peripheral blood CD4^+^ T cells from ART-suppressed HIV^+^ individuals presents a challenge for quantifying viral protein expression, even with sensitive assays (Falcinelli et al., 2019). To further assess performance of our new approach, we activated peripheral blood CD4^+^ T cells from ART-suppressed HIV^+^ individuals and quantified p24 protein production by IP-Simoa. Figure 3A represents a single ART-suppressed individual with low-level basal p24 expression in peripheral blood CD4^+^ T cells in the absence of T-cell stimulation and without IP (mean = 0.023 pg/mL, *n* = 3). *Ex vivo* exposure of CD4^+^ T cells from this donor with anti-CD3/anti-CD28 conjugated beads for 3 days resulted in a significant 15-fold increase in p24 to an average of 0.34 pg/mL (*n* = 3, *p* < 0.05) even in the absence of IP. IP-Simoa resulted in approximately 5-fold further increase in p24 concentration in the cell lysate (1.65 pg/mL; *n* = 3) from the same donor. This increase in p24 upon 5-fold volume reduction was statistically significant (*p* < 0.001; Figure 3A) and indicates that virally produced gag p24, like recombinant p24, was efficiently immunoprecipitated and eluted with this IP procedure. No significant difference was observed between unstimulated p24 levels between non-IP and IP methods with this donor, potentially attributed to the low frequency of transcriptionally and translationally active HIV infected cells in the unstimulated sample and variable expression of p24 across HIV proviral genomes. In our experience, p24 detection in resting cells from ART-suppressed donors is infrequent (data not shown). To extend this work, we evaluated anti-CD3/anti-CD28 bead stimulation of CD4^+^ T cells isolated from blood of five additional HIV^+^, ART-suppressed individuals and observed a similar pattern of volume-proportional increases following IP-Simoa vs. without IP Simoa (*p* < 0.01) (Figure 3B). p24 values were below assay limits in the flow-through following protein capture on beads, indicating the IP method efficiently captured the expressed p24. Next, we identified a subset of ART-suppressed HIV^+^ samples lacking measurable p24 by traditional direct, non-IP Simoa methods following anti-CD3/anti-CD28 bead stimulation. We assessed the ability for the IP-Simoa method to enrich and quantify p24 following stimulation and compared results to samples from HIV-negative donors. Following T-cell reactivation and IP-Simoa, low but measurable p24 levels were detected in all HIV^+^ samples, whereas HIV-negative samples remained below assay limits (Figure 3C). Donor cells that have not yielded p24 under any of our assay conditions tested to date have also been encountered (data not shown).

We evaluated inducible p24 levels following treatment with the well-studied latency-reversing agent and HDACi, VOR. Measurable p24 levels were observed in three out of five HIV^+^, ART-suppressed samples treated with 1 μM VOR for 72 h (Figure 3D) and were lower than anti-CD3/CD28 bead-stimulated samples (data not shown), consistent with previous findings (Figure 3D; Wu et al., 2017; Falcinelli et al., 2019). Seventy-two hours was chosen to assay for comparison to anti-CD3/anti-CD28 stimulation. Shorter or longer time periods will likely induce different levels of protein and can be explored in future studies, along with the use of different latency-reversing agents. Use of a non-specific kinase inhibitor, staurosporine, to non-selectively kill cells during the stimulation period, prior to protein immunocapture was also evaluated. Under these conditions, p24 decreased dramatically following 1 μM staurosporine treatment of cells prior to cell lysis and immunocapture of p24 (Figure 3E), indicating IP-enriched p24 was generated from live cells.

### Measurement of p24 From Rectal Pinch Biopsies

**FIGURE 4:**
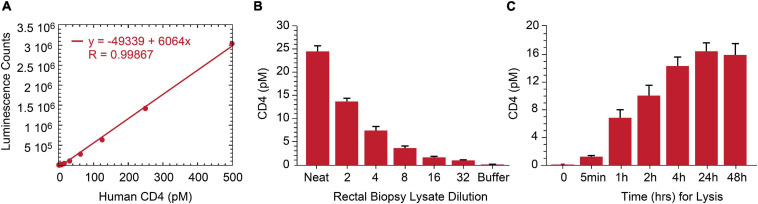
Development of human CD4 protein assay and application to rectal biopsy lysate characterization. **(A)** Signal in the ELISA was proportional to the CD4 protein level over a broad standard curve concentration range from 0 to 250 pM. **(B)** Assay specificity was shown with CD4 signal linearly decreasing following rectal biopsy sample lysate serial dilution. **(C)** Twenty-4 h of biopsy soaking while mixing in lysis buffer yields complete CD4 protein release from rectal biopsy.

As lymphoid tissue is a reservoir for persistently infected HIV cells (Wong and Yukl, 2016; Thornhill et al., 2019), we assessed p24 measurement by IP-Simoa in gut-associated lymphoid tissue (GALT) from ART-suppressed, HIV^+^ individuals. Our original approach necessitated dissociation and isolation of single-cell suspensions from intact rectal tissue biopsies to overcome high matrix background effect (Wu et al., 2021). This method is quite laborious and low-throughput. To reduce procedure complexity, we evaluated if soaking intact tissue biopsy in 1% Triton X-100/Simoa lysis buffer would sufficiently dissociate tissue and yield high recovery of the cells of interest. As HIV predominantly infects CD4^+^ T cells, we selected CD4 protein as a surrogate biomarker for the primary target cell population and developed at CD4 ELISA to assess efficiency of tissue cell lysis. A standard curve using recombinant protein was generated and demonstrated robust linearity quantifying CD4 protein in the range of 0–250 pM (Figure 4A). The assay was tested with both peripheral CD4^+^ T cells and normal human rectal tissues. CD4 concentration was measured in five ART-suppressed donor’s PBMC and was found to be correlated with CD4 counts in samples using equivalent cell numbers per test (*r* = 0.944, *p* < 0.01, *n* = 5) (Data not shown). CD4 protein was also reliably quantified in HIV-negative rectal biopsy after soaking in 1% Triton X-100/Simoa lysis buffer and decreased linearly upon sample dilution (Figure 4B). Time-course studies revealed CD4 levels plateau at some time greater than 4 h of soaking in lysis buffer [signal increases another 10% with overnight soaking with no significant difference between 24 and 48 h (Figure 4C)]. No detectable CD4 protein was found in pellet following resuspension in the lysis buffer and homogenization (data not shown). Thus, 24-h soaking was selected as an optimal lysis time for rectal tissue dissociation based on CD4 measurements and use overnight incubation for our procedures.

**FIGURE 5:**
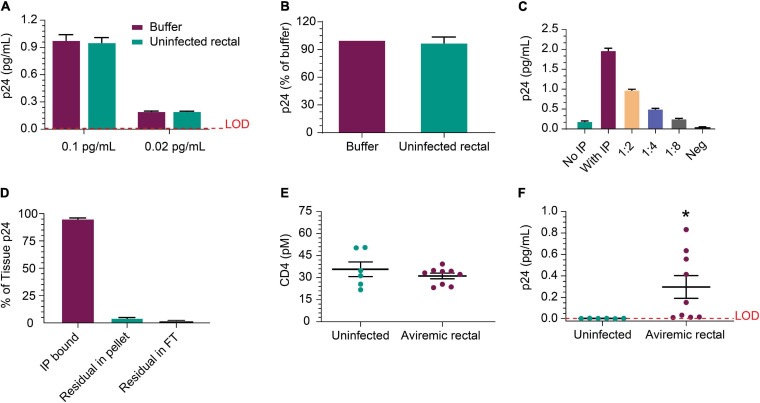
Detection of p24 protein from rectal pinch biopsies. **(A)** Recovery of recombinant p24 spiked into biopsy lysate at 0.02 and 0.1 pg/mL, respectively, is comparable to recovery from buffer as the matrix after IP concentration. **(B)** Recovery was comparable between buffer and uninfected rectal lysate matrix when using p24 protein recovered from a viremic donor-derived sample as the protein source for spike-in. **(C)** Cells isolated from a viremic rectal biopsy were lysed and run in standard p24 Simoa or IP-Simoa. The p24 was enriched by the volume reduction during IP and shows linear signal after dilution supporting signal specificity. **(D)** Reprocessing of rectal biopsies (*n* = 3) for p24 after lysis revealed that a single extraction yielded >96% of the p24 contained within the biopsy. There was no detectable p24 in solution after IP, indicating complete capture of p24 onto the beads. **(E)** Comparing measured CD4 protein from HIV-negative and HIV-positive, ART-suppressed rectal biopsies showed no significant difference in CD4 level, implying that similar numbers of CD4^+^ cells were present and lysed from each group. **(F)** Using the same lysates as in **(E)**, p24 was measurable in several of the HIV^+^ donor biopsy samples (five of nine) after IP-Simoa and not in biopsies from HIV-negative donors (*p* < 0.05, *n* = 6). See Materials and Methods for statistical analysis.

Next, we evaluated whether p24 could be released from HIV^+^ biopsies and efficiently immunoprecipitated from the tissue matrix. Figure 5A shows IP recovery was comparable between buffer and HIV-negative rectal lysate following the addition of recombinant p24 in the sample at either 0.1 or 0.02 pg/mL. Using p24 isolated from viremic rectal biopsy tissue was recovered at similar levels after inoculation of either buffer or uninfected rectal lysate (Figure 5B). These data indicate that rectal tissue lysate matrix has no detrimental impact on capture and detection of recombinant p24 and that p24 from donor-derived samples can be detected as well. Using cells dissociated from a rectal pinch biopsy from a viremic donor, p24 was detected well, even without IP and increased ∼10-fold upon 10 × concentration of volume by IP; signal decreased linearly with dilution (Figure 5C). This indicates that p24 recovered from a rectal biopsy can be efficiently measured. Using additional viremic biopsies (*n* = 3), we used the direct biopsy lysis method to verify the release of the p24 and quantitate the completeness of p24 release. After soaking and removal of supernatant, we re-ran the assay on the residual tissue and found ∼4% of the original p24 concentration was recovered upon reprocessing, showing high but incomplete recovery by a single cycle. No p24 was detected in the flow-through after IP (Figure 5D). To extend these studies, additional rectal biopsies were obtained from six uninfected and nine ART-suppressed, HIV^+^ individuals with undetectable viral load (<50 copies/mL). CD4 protein level trended higher in uninfected group but not significantly when compared to the HIV-infected group and could be a product of sample preparation (see section “Participants” in “Materials and Methods”) (Figure 5E). Despite slightly higher CD4 protein level, the HIV^+^ samples had significantly (*n* = 9, *p* = 0.041) higher p24 than HIV-negative (Figure 5F). We also initiated studies to assess whether CD4 ELISA values correlated with CD4 cell counts. Collectively, p24 was reliably detected after soaking in lysis buffer and IP-Simoa from five of nine samples (~56%) and near the lower LOD for the other four samples.

### IP-Simoa Assay for SIV p27 Protein Measurement

**FIGURE 6:**
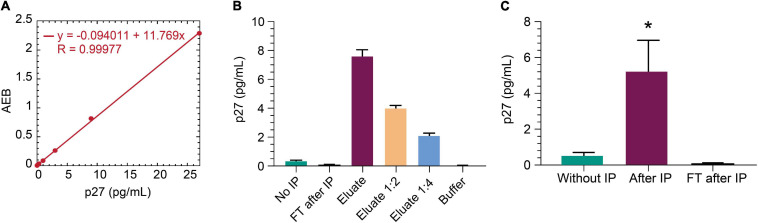
IP-Simoa assay for SIV p27 protein measurement. **(A)** SIV p27 Simoa assay standard curve. Linear signal response for recombinant p27 from 0 to 28 pg/mL. **(B)** Recombinant p27 spiked into uninfected rhesus monkey rectal biopsy (prepared by soaking in lysis buffer) and run with IP Simoa showed enriched p27 signal proportional to the sample volume reduction (20-fold) and expected reduction of signal upon dilution indicating assay specificity. **(C)** p27 was enriched significantly from the lysate of viremic, SIV^+^ monkey rectal biopsy (*p* < 0.05, *n* = 4). No detectable p27 was observed in the flow through after IP demonstrating high efficiency of p27 IP from biopsy lysate matrix. See Materials and Methods for statistical analysis.

The non-human primate SIV model is an important model for studying HIV cure (Evans and Silvestri, 2013). SIV gag p27 protein is a relevant viral biomarker of SIV persistence, as outlined above for HIV gag p24 protein. To that end, we sought to determine whether the IP-Simoa p24 assay could be adapted for SIV p27. Anti-p24 beads were selected for p27 immunocapture as this p24 antibody efficiently binds both HIV gag p24 and SIV gag p27 (data not shown). An SIV p27-specific antibody was biotinylated and used for detection in the Simoa assay. Figure 6A shows the standard curve for recombinant p27 (1:3 dilution; concentration range from 27 to 0.02 pg/mL). Assay LOD was determined to be ∼0.02 pg/mL, similar to HIV gag p24 Simoa assay. To assess if IP-Simoa can enrich for SIV p27, we spiked recombinant p27 into lysates prepared from SIV-negative rhesus rectal biopsy and measured p27 in the bead and flow-through fractions. Only the immunobead fraction had quantifiable levels of p27, and protein concentration decreased linearly upon serial dilution (Figure 6B). We extended these studies to rectal biopsies (*n* = 4) obtained from a viremic rhesus monkey (plasma viremia at 9,300 copies/mL) infected with chimeric simian–human immunodeficiency virus (ENV162P3 SHIV; Harouse et al., 2001). As above, most p27 was detected in the immunocaptured fractions with little to no measurable p27 in the flow-through (Figure 6C). Thus, the new IP-Simoa methodology is applicable for the sensitive detection of both SIV gag p27 and HIV gag p24 proteins.

## Discussion

As shown recently (Wu et al., 2017, 2021), the detection of low-abundant viral proteins from HIV-expressing cells from blood or lymphoid tissue of HIV infected, ART-suppressed individuals is possible but still presents challenges. Various approaches have been described for concentrating cell lysates and enriching analytes (Wood, 1991); however, these approaches can fall short as increasing concentration and sample viscosity may interfere with assay signal. Sample dilution may overcome any matrix interference but is less desirable when high sample concentration is needed for detecting low-abundant analytes. Thus, sample preparation is a fine balance between having the analyte of interest as concentrated as possible without raising matrix and assay background to untenable levels. Furthermore, consideration must be given to the compatibility of the biological sample type and the analyte detection methods.

Here, we apply a novel immunocapture and sensitive ELISA approach (IP-Simoa) for the maximal and specific measurement of HIV and SIV gag proteins from biological samples, particularly at low concentrations. In this study, we have made strides in more clearly delineating between positive and negative samples by immunocapturing the maximal amounts of protein from the sample onto beads, removing non-specific interference from sample matrix through washing, eluting in such a manner as to retain antibody recognition of the analyte, and resuspending in a final volume such that all sample is loaded onto the instrument for quantitation (Figure 1). We experimentally determined that TFA, a common acid used in spectrometry-based proteomics to dissolve cells/tissues and achieve highly efficient protein extraction, was optimal for efficient recovery of viral protein from beads. We then applied the new method to measure both recombinant p24 (or p27) spiked into biological samples at very low concentrations (Figures 2, 6), as well as p24 induced from samples derived from virally suppressed CD4^+^ T cells (Figure 3) or viremic rectal pinch biopsy (Figure 5). Our findings suggest a range of protein detection (0.001–3 pg/mL) with recovery efficiency of 85–95%. As the focus of our study was on detecting low levels of p24, we did not extensively investigate the upper range of detection beyond 3 pg/mL; however, we believe this is feasible. Detecting higher concentration of p24 (or p27) can be achieved either by direct Simoa method (ULOD 30 pg/mL; Quanterix, Inc.), or alternatively, conducting independent studies using the IP method to determine optimal bead concentrations for capturing large amounts of protein.

Several tests were carried out to understand the reproducibility of the assay and to ascertain if enrichment reagents might interfere with p24 quantification. In our studies, both intraday and interday CV of spiked-in, low-concentration p24 protein into buffer followed by IP and Simoa is below 15%. The p24 recovery for both intraday and interday is from 85 to 95%. The high recovery is probably due to the saturating amount of high-affinity antibody used during IP, coupled with overnight incubation for capture, and efficient elution conditions. Several studies suggest the lack of interference of the enrichment antibody or IP beads in p24 quantification. For example, we achieved near 100% protein recovery after volume adjustment using the IP-Simoa (Figure 2), suggesting the presence of antibody or beads, if released into the eluate, did not interfere with protein measurement on the Quanterix instrument. In addition, there was no measurable signal in blank IP samples, which lacked spiked p24 (data not shown), further strengthening the notion that either IP beads are not retained in the eluate, or the few beads that may be eluted do not interfere in protein quantification.

With optimized conditions, the p24 IP Simoa was successfully employed to measure p24 protein derived from ART-suppressed, HIV-positive donor cells that had been stimulated *ex vivo* to reactivate latent virus. From these, p24 was measured after enrichment, and levels were significantly higher than that of non-enriched lysate and uninfected controls (Figure 3). These findings indicate that the assay is specific and works to detect p24 derived from a variety of different donors. This assay has shown value not only for studying p24 from suppressed donor samples that respond robustly to stimulus, but also for weakly inducible donors. As p24 without enrichment is not detected from some donors after stimulation, this limits the number of donors whose cells can be used in *ex vivo* studies that look at protein production. By IP enriching for p24, we were able to expand the number of donors whose cells can be used for such studies. It has been shown that p24 can be detected from a single cell using the digital ELISA format (Passaes et al., 2017). However, the ability to detect p24 in induced samples that were previously undetectable by Simoa with the incorporation of a p24 IP step would suggest that there are p24-producing cells that are still not detectable by standard Simoa procedures. This likely reflects the heterogeneity of the proviral pool with various protein levels inducible from latently infected cells. The method has also been shown to more robustly detect p24 induced by using a weak LRA, such as VOR over no enrichment (Figure 3). Starting with cells that respond robustly to strong stimulus, one should to be able to study LRAs of various mechanisms over multiple donor-derived samples. These advances should provide a valuable tool for studying treatment efficacy *ex vivo* with latency-reversing agents or selective kill agents with real-world virus and donor cells. Measuring the kinetics of latency reversal along with washout experiments for various molecules and mechanisms should provide insight into potential dosing schema for *in vivo* testing.

As the gut-associated lymphoid tissues are an important HIV reservoir (Wong and Yukl, 2016; Thornhill et al., 2019), and it is possible to collect rectal tissue pinch biopsies in the context of a clinical program, the IP Simoa has the potential to be used as a pharmacodynamic biomarker for studying the efficacy of HIV latency-reversing agents, therapeutic efficacy of cure-focused agents, rebound after analytical treatment interruption, or to study the possible mechanisms of HIV persistence. The simplified lysis procedure coupled with the enhanced sensitivity and selectivity of the IP Simoa may translate well into a clinical trial setting. Not every suppressed patient’s rectal pinch biopsy has shown a positive p24 signal in our preliminary studies, so understanding the factors influencing this and longitudinal sampling reproducibility are important for applications as a biomarker of HIV persistence. Having these enhanced protocols in hand would allow for efficient study of these questions with a proper cohort of donors. Our finding that rectal biopsies lysed directly in Triton X-100 lysis buffer instead of from cells dissociated from rectal biopsy has simplified sample preparation and likely improved protein tissue extraction efficiency. This simplification in the processing procedure makes this combined assay more robust for potential clinical application. While we use CD4 protein as a surrogate marker for cell lysis, the value of this readout as a normalization factor is unknown. Because of the scarcity of p24-producing cells in a suppressed donor, one would need to assume uniform distribution of these cells relative to the CD4^+^ cells in order to reliably normalize to it. Further insights are needed before employing CD4 measurements as a normalization factor. An additional area of interest would be to assess p24 produced after induction of tissue-derived cells. Understanding the translationally competent reservoir lymphoid tissues (as compared to blood-derived cells) should prove enlightening.

Future directions include use of this method as both a clinical biomarker and a non-clinical tool for studying expression of viral antigen upon *ex vivo* stimulation. In order to facilitate both clinical and research applications, automation of the procedure from the current manual IP done into a 96-well plate-based assay will be very enabling, and efforts are currently underway in our laboratory. Such an automated assay using patient-derived PBMCs will be powerful for studying latency-reversing agents or selective kill approaches in dose response across multiple donors. The demonstration of the IP Simoa assay to enable detection of p24 from *ex vivo*–stimulated CD4^+^ T cells from donors who were not previously detectable will enable broader exploration of different donors to help ensure breadth of coverage of novel therapeutics. Further adaption of the assay should also allow for similar studies aimed at effector cell–mediated killing, such as ADCC-type approaches with broadly neutralizing antibodies leveraging patient-derived virus as well as autologous effector cells. We feel that these studies across as many donors as is feasible are necessary to help understand breadth of applicability of different potential therapies. Genetic variation as well as patient-specific variations will undoubtedly be critical to understand as fully as possible prior to initiation of clinical studies. Clinical validation of the IP method will allow the use of viral protein measurements as an endpoint in determining efficacy of novel therapeutics, which rely on the expression of viral proteins. Emerging data are starting to show correlates between p24 expression and other markers of immune status (Wu et al., 2021). Expanding the use of the p24 measurements to further build on these early observations will be critical for understanding how viral protein production impacts pathophysiology and immune status/function in ART-treated patients. For further clinical applications, examining p24 induced from donor samples can be utilized for studies, such as looking at p24 expression magnitude, p24 capable reservoir size, and other potential assays.

In summary, we have developed novel, specific, and sensitive combined IP Simoa p24 and p27 immunoassays. The IP Simoa method has been validated with HIV-negative donors, *ex vivo*–reactivated CD4^+^ T cells from HIV^+^ donors, and rectal biopsies from viremic and aviremic patients and non-human primates. The advantage of combined IP Simoa assay is that all analytes from a given sample will be enriched from the lysate volume, regardless of lysis volume and instrument limitation, and separated from all other cellular proteins that contribute to assay background. We feel that the assay should be amenable to automation and will be beneficial to both clinical and research applications for HIV cure efforts.

## Data Availability Statement

All datasets generated for this study are included in the article/Supplementary Material, further inquiries can be directed to the corresponding author/s.

## Ethics Statement

The studies involving human participants were reviewed and approved by the IRB. The patients/participants provided their written informed consent to participate in this study. The animal study was reviewed and approved by the IACUC. Written informed consent was obtained from the owners for the participation of their animals in this study.

## Author Contributions

GW, DH, BH, and PZ conceptualized and designed the experiments. GW, CC, and QH performed the experiments and generated the data. GW, BH, and PZ wrote the manuscript. All authors edited and approved the submitted version.

## Conflict of Interest

GW, CC, QH, DH, BH, and PZ were employed by Merck & Co., Inc.

## Supplementary Material

Supplementary Figure 1 IP-Simoa assay comparing HIV-negative biopsies to buffer. Tissues from five HIV-negative donors with three replicates each donor were tested alongside buffer-only samples. After the procedure was complete, the buffer only samples were indistinguishable from the tissue samples indicating that there is no background contribution of the matrix to the readout.
