# Supplementary figures and images for: Improved Detection of HIV Gag p24 Protein Using a Combined Immunoprecipitation and Digital ELISA Method
Source: Front Microbiol. 2021 Mar 16;12:636703. doi: 10.3389/fmicb.2021.636703 (PMC8007784; doi:10.3389/fmicb.2021.636703)

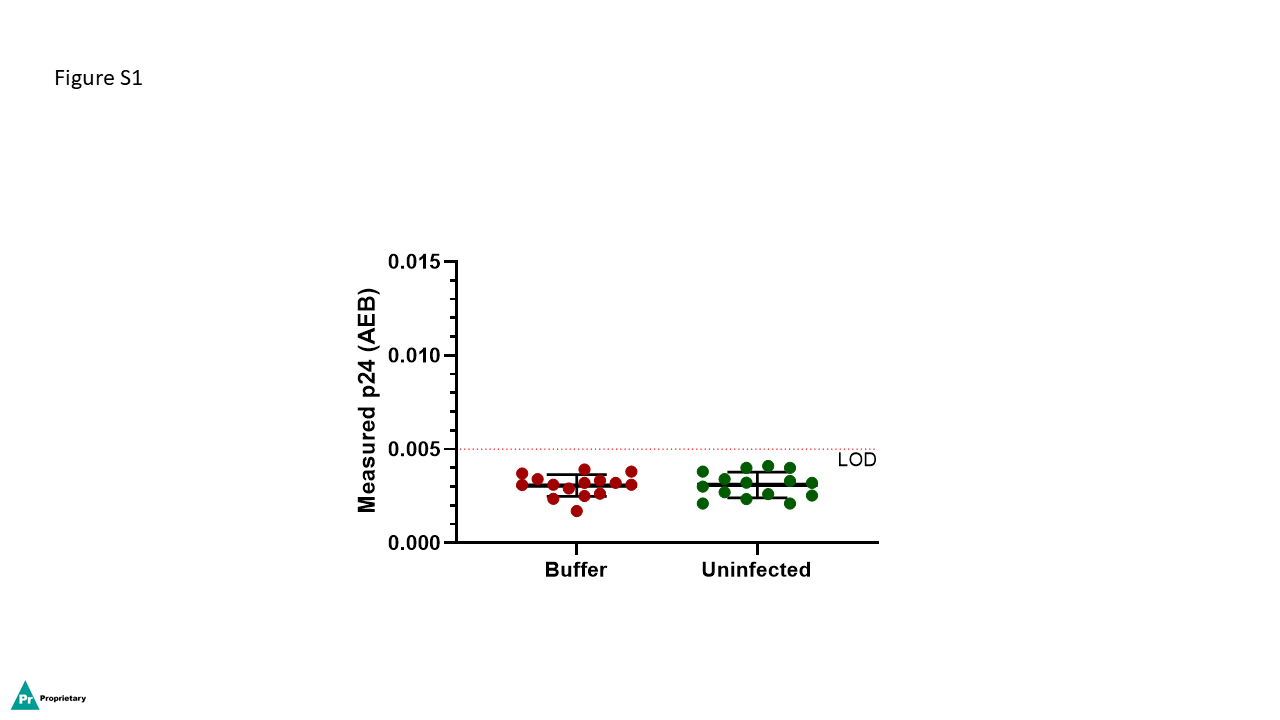

Supplement: Supplementary Figure 1 — IP-Simoa assay comparing HIV-negative biopsies to buffer. Tissues from five HIV-negative donors with three replicates each donor were tested alongside buffer-only samples. After the procedure was complete, the buffer only samples were indistinguishable from the tissue samples indicating that there is no background contribution of the matrix to the readout. [file Image_1.TIF]
